# Exploring the impact of age and gender on retinal and choroidal thickness and vascular densities: a comprehensive analysis

**DOI:** 10.1186/s40942-024-00619-4

**Published:** 2025-03-31

**Authors:** Fariba Ghassemi, Morteza Karimi, Farhad Salari, Kia Bayat

**Affiliations:** 1 Eye research center, Farabi Eye Hospital, Tehran University of Medical Sciences, Qazvin Square, Tehran, 1336616351 Iran; 2 Retina & Vitreous Service, Farabi Eye Hospital, Tehran University of Medical Sciences, Tehran, Iran

**Keywords:** Choriocapillaris, Choroidal vascular density, Choroidal vascular index, Deep capillary plexus, Inner retinal thickness, Middle retinal thickness, Outer retinal thickness, Retinal thickness, Superficial capillary plexus, Vascular density

## Abstract

**Purpose:**

To evaluate the significance of age and gender on macular retinal and choroidal thicknesses (RT and CT) and related vascular density (VD) using optical coherence tomography angiography (OCTA) in a wide spectrum of normal participants.

**Methods:**

This was a cross-sectional study of 435 eyes of 234 normal healthy children and adults aged 5–97 years old who performed macular 3 × 3 mm scans using Optovue RTVue OCTA.

**Results:**

A statistically meaningful relationship was detected between age and macular layered VD and RT parameters. The middle retinal thickness (MRT) experiences minimal alterations throughout the lifespan. The mean foveal superficial capillary plexus density (SVD), deep capillary plexus density (DVD), and parafoveal SVD had the most density between the 20–40 years and tended to decrease to lowest point by the seventies. Foveal choriocapillaris vascular density (CVD), parafoveal CVD and parafoveal DVD had a decreasing course during the life course. There was no significant difference in choroidal vascular index between age groups. FAZ revealed relatively insignificant minor changes across age groups. The male participants had higher VD than the female participants in all measured parameters, except for parafoveal DVD.

**Conclusion:**

RT of different layers as well as the whole retina is known to be influenced by age while the MRT experiences minimal alterations throughout the lifespan. The VD of male participants exceeded that of female participants across all measured parameters, except parafoveal DVD. Differential retinal and choroidal vascular plexuses have different course during a man’s life.

## Introduction

The macula is a highly specialized area that serves multiple visual functions including visual acuity, color and shape perception, and stereopsis. It is responsible for the 18 degrees at the center of the visual field [1]. The macula is often affected by various ocular, neurological and systemic diseases. Some of these diseases can be typically degenerative in nature leading to neuronal loss and thinning of the entire macula and/or different layers within it or cause an increase in the thickness of macular layers [2].

Optical coherence tomography (OCT) as a noninvasive and objective method, is a widely accepted technique for obtaining high-resolution images of the retina offering data comparable to that of a histological analysis [3]. Its strong repeatability and reproducibility make it a reliable technique that could be used in both adult and pediatric populations [4]. In addition, precise quantitative measurements aid in the diagnosis and monitoring of the progression or response to treatment of different retinal pathologies.

It is essential to have a comprehensive understanding of the condition of normal eyes before their implementation in clinical settings. Furthermore, several previous investigations have ascertained that the retinal and choroidal thicknesses (RT and CT), and the respective vascular density (VD) fluctuate in accordance with both age and gender. A study conducted on 100 healthy individuals, aged between 6 and 79 years, demonstrated a significant decrease in RT with an annual reduction of 0.53 μm per year [5]. In another study, RT exhibited a correlation with age across all regions of the macula [6]. The decrease in RT observed throughout all areas during the aging potentially signifies degenerative modifications that occur with advancing age. Conversely, the thickening of the fovea in younger age groups likely denotes alternative aging mechanisms, such as the potential accumulation of extracellular or intracellular debris [6]. Conversely, some studies found no significant relationship between macular thickness and age [7, 8]. Collectively, it seems that the results are conflicting in this regard. Hence, we conducted this study to establish a standard database for RT and CT and corresponding VD(s) in a healthy Iranian population, as well as to evaluate the influence of age and gender on these factors. Our objective was to assess the changes in layered retinal and choroidal thicknesses, as well as the associated vascular densities, on a decade-by-decade basis.

## Materials and methods

This cross-sectional retrospective cohort study was conducted at the Retina Department of Farabi Eye Hospital between June 2022 and June 2023. Both healthy male and female participants of all ages were included in the study.

This study followed the tenets outlined in the Declaration of Helsinki protocol and was approved by the Tehran University of Medical Sciences Review Board (IR.TUMS.VCR.REC.1397.1054) for the children and adolescents and we added the data of some other adult’s data from medical records or archived pictures of optical coherence tomography angiography (OCTA). All participants, or their authorized guardians, provided written/oral informed consent to participate in this study. The mandatory criteria for inclusion consisted of a best-corrected visual acuity (BCVA) of 20/20, a refractive error ranging from − 1D to + 1D spherical equivalent (SE), and an intraocular pressure less than 21 mmHg. Individuals with issues pertaining to blinking, a history of ocular surgery, as well as systemic or ocular diseases were excluded from this study. In our earlier reports, we assessed the retinal and choroidal thicknesses, as well as the vascular densities, in four distinct categories of both adult and pediatric eyes. In this study, our objective was to assess the changes in layered retinal and choroidal thicknesses, as well as the associated vascular densities, on a decade-by-decade basis. All participants underwent imaging via the AngioVue OCTA system version 2018,0,0,18 (Optovue RTVue XR Avanti, Optovue Inc., Freemont, California, USA) during the morning session of the workday. The imaging was done from June 2022 up to Oct 2022.

Specific measurements were conducted based on the subsequent definitions: the inner RT (IRT) was signified as the distance between the internal limiting membrane (ILM) and the outer boundary of the inner plexiform layer (IPL). The middle RT (MRT) was established as the distance from the outer boundary of the IPL to the outer limit of the outer plexiform layer (OPL). Meanwhile, the outer retinal thickness (ORT) was characterized as the distance between the outer margin of the OPL and the outer edge of the retinal pigment epithelium (RPE)-Bruch’s membrane complex (BRM). For this investigation, the RT was calculated for diverse sectors within the central 3 mm diameter of the macula, adhering to the Early Treatment Diabetic Retinopathy Study (ETDRS) grid. The fovea location was automatically determined. The central circle of 1.0 mm was considered as the foveal region. The parafoveal area was defined as circle with a diameter of 1–3 mm width circle surrounding the foveal area.

The automation of the segmentation of superficial capillary plexus (SCP), deep capillary plexus (DCP), and choriocapillaris plexus (CCP) were determined by employing the incorporated software algorithm. The upper and lower boundaries of SCP were determined to be 3 μm below the inner limiting membrane (ILM) and 15 μm beneath the inner plexiform layer (IPL), respectively. The boundaries of DCP were located between 15 μm and 71 μm below the IPL. The CCP was defined as the region situated between 15 and 45 μm below the Bruch’s membrane. The software employed calculated the VD as the relative flow density (expressed in percentage) in the binary reconstructed images in the capillary plexuses (superficial capillary plexus vascular density: SVD, deep capillary plexus vascular density: DVD, choriocapillaris plexus vascular density: CVD). The study calculated the VD in various sectors as the complete image, fovea, parafovea, and subsegmental areas including temporal, superior, nasal, and inferior parafoveal area, based on the ETDRS grid. Additionally, to assess the vascularity of the choroid, the choroidal vascular index (CVI) was computed by dividing the luminal area (LA) by the total circumscribed sub-foveal choroidal area, as previously outlined [9]. The subfoveal choroidal thickness was assessed automatically using a predetermined method by Optovue RTVue- AngioVue OCTA, measuring from the outer segment of Bruch’s membrane to the sclerochoroidal interface at the fovea.

In the calculation for the sample size of the study, a minimum of 208 participants was obtained (26 individuals in each group), considering a 95% confidence interval, a 10% change in VD, and a population proportion of 50%. Statistical analysis was done using SPSS software version 27 from SPSS, Inc., Chicago, IL, USA. All normally distributed data was reported as the mean with standard deviation and non-normally distributed data was presented as the median with an interquartile range. Considering the intra-eye correlations, the generalized estimating equation model (GEE) was applied to show the differences between age groups. Pairwise comparisons were corrected using the Sidak method. A *P*-value below 0.05 indicated statistical significance.

## Results

**Fig. 1:**
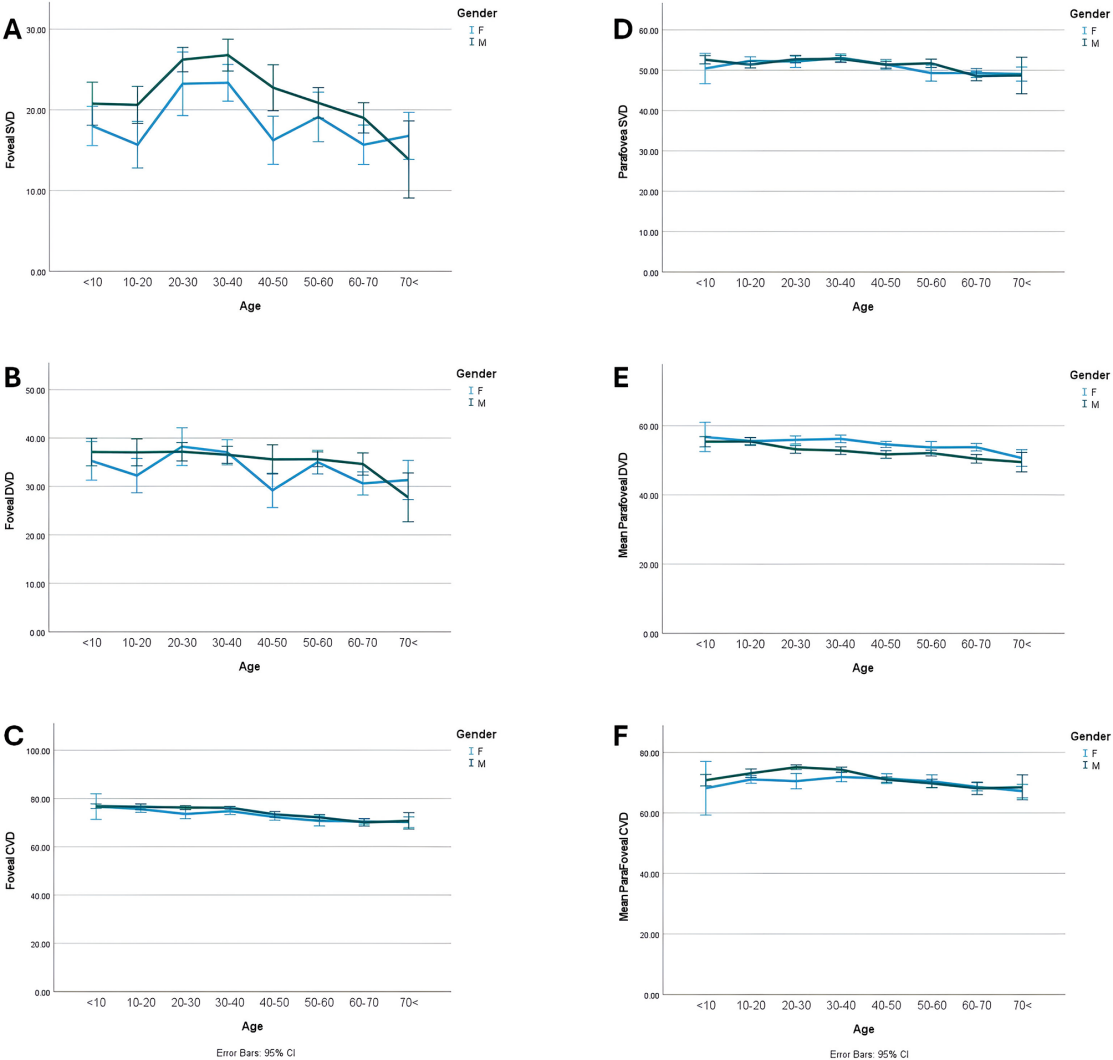
Foveal and parafoveal vascular densities of normal healthy people in both genders in different age groups. The figure reveals pronounced gender-based disparities in foveal SVD and DVD measurements. In contrast, parafoveal SVD and DVD exhibit negligible intergender differences. **A.** Foveal SVD is higher in males across all age groups compared to females except in the final age group, following similar trends of increase and decrease. **B.** Foveal DVD in females shows a more variable course than in males. **C.** Foveal CVD in the choroid follows a similar course and magnitude in both males and females. **D.** Parafoveal SVD exhibits comparable trends and values in both groups, with minimal changes over time. **E.** Parafoveal DVD demonstrates a more stable course than foveal DVD. Except in the youngest age group, females generally have higher density in this region. **F.** Although parafoveal CVD is higher in males in the first age group, after age 40, the values become comparable between sexes. *Abbreviations*: SVD, superior vascular density; DVD, deep vascular density; CVD, choroidal vascular density

**Fig. 2:**
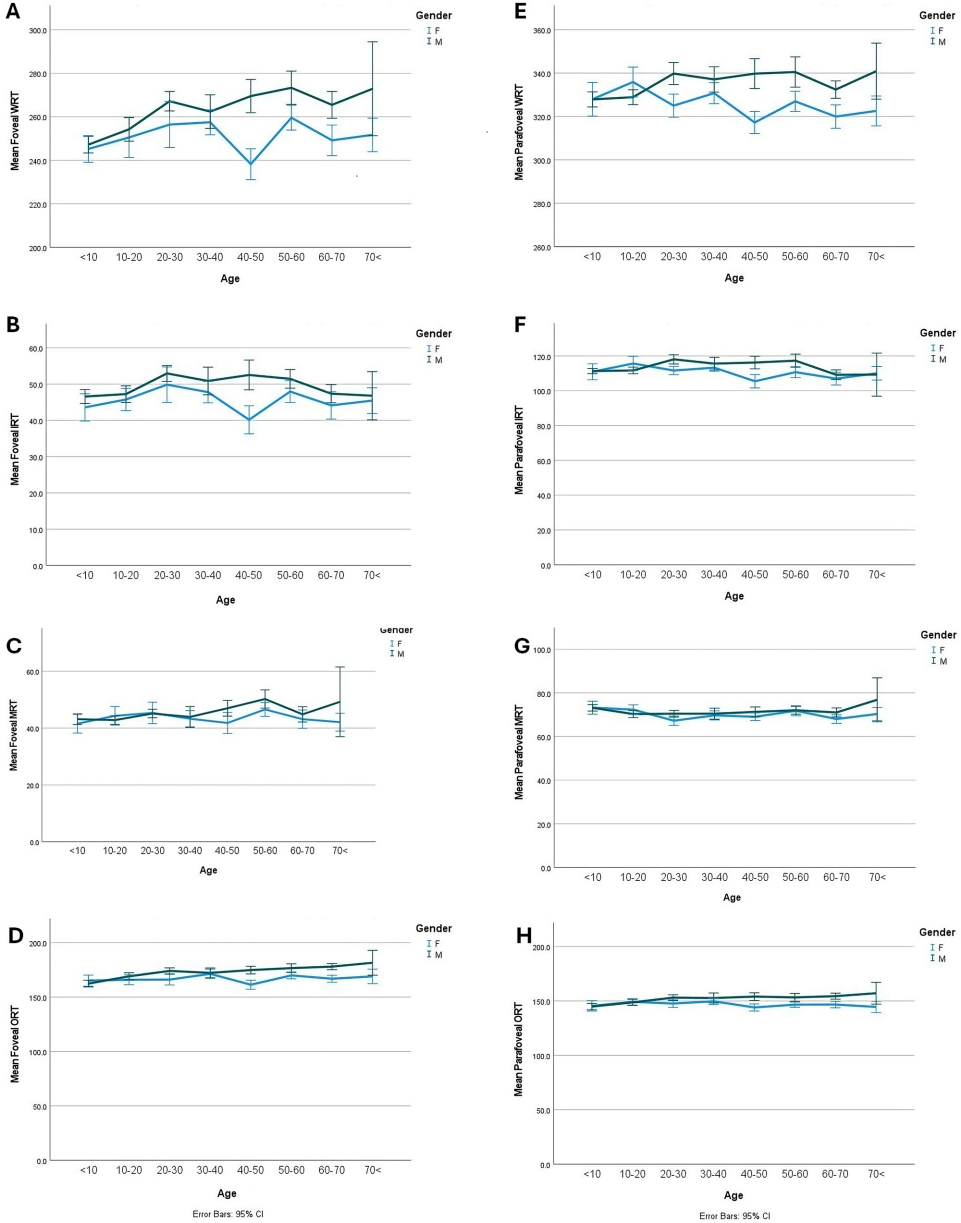
Foveal and parafoveal retinal thickness of normal healthy people in both genders in different age groups. Overall, males exhibit greater foveal retinal thickness compared to females. This gender difference persists in parafoveal RT measurements across all age groups except the youngest cohort. However, when examining individual retinal layers, thickness variations between genders become negligible. **A.** Mean foveal WRT is higher in males compared to females. Females exhibit greater variability over time. **B.** Changes in foveal IRT follow a similar pattern to WRT (as described in A). **C.** In foveal MRT, the first three age groups show comparable trends and values between sexes, but after age 40, thickness decreases in females. **D.** In foveal ORT, thickness is slightly lower in females than in males, except in the youngest age group. **E.** For parafoveal WRT, males have higher values than females in all age groups except the second. **F.** In parafoveal IRT, trends and values are similar between sexes, but females have lower values except at the extreme ages. **G.** In parafoveal MRT, values and trends are comparable across all age groups except the oldest, where differences emerge. **H.** In parafoveal ORT, the first two age groups show comparable values between sexes, but thereafter, females have slightly loweramounts. *Abbreviations*: IRT, inner retinal thickness, MRT, middle retinal thickness; ORT, outer retina thickness; WRT, whole retinal thickness

**Fig. 3:**
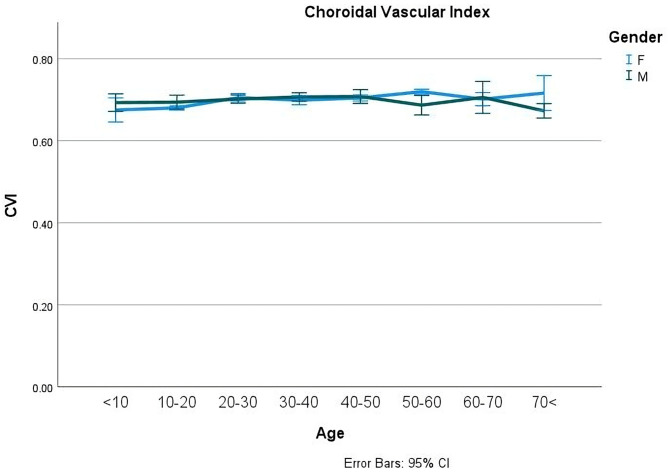
Choroidal vascular index (CVI) of normal healthy people of both genders in different age groups. The choroidal vascularity index (CVI) exhibited minimal changes across age groups. Slight but notable differences were observed at the extreme age ranges and particularly between 50-60 years, where females exhibited marginally higher values than males

In this study, a total of 234 participants (435 eyes) were included, with females accounting for 41.9% of the participants. The study results are presented in Figs. 1, 2 and 3; Tables 1, 2, 3 and 4, offering a comprehensive overview.

**Table 1:** Foveal and parafoveal vascular densities in healthy subjects in 8 age groups. Abbreviations: SVD: superficial vasclar density; DVD, deep vascular density; CVD, choroidal vascular density; CVI, choroidal vascular index and FAZ, foveal avascular zone

		Group1	Group2	Group3	Group4	Group5	Group6	Group7	Group8	
	Total (369 Eyes)	< 10 (26 Eyes)	10–20 (39 Eyes)	20–30 (49 Eyes)	30–40 (56 Eyes)	40–50 (51 Eyes)	50–60 (53 Eyes)	60–70 (70 Eyes)	> 70 (25 Eyes)	*P*
**Foveal** **SVD**	20.51 ± 7	20.23 ± 5.42	18.34 ± 5.88	25 ± 6.29	25.31 ± 5.65	19.68 ± 7.79	20.21 ± 5.84	17.29 ± 6.57	15.96 ± 5.73	< 0.001
**Parafoveal SVD**	51.15 ± 3.22	52.18 ± 2.51	51.81 ± 1.9	52.47 ± 2.69	52.92 ± 2.31	51.4 ± 2.51	50.79 ± 3.61	48.94 ± 3.31	48.94 ± 3.83	< 0.001
**Foveal DVD**	34.68 ± 6.94	36.77 ± 5.78	34.85 ± 6.94	37.62 ± 6.5	36.78 ± 5.41	32.6 ± 8.55	35.41 ± 4.61	32.58 ± 7.08	30.34 ± 7.55	0.001
**Parafoveal DVD**	53.43 ± 3.52	55.64 ± 3.25	55.49 ± 2.26	54.28 ± 3.05	54.26 ± 3.3	53.05 ± 2.86	52.7 ± 2.98	52.16 ± 3.73	50.32 ± 4.4	< 0.001
**Foveal CVD**	73.34 ± 4.05	76.75 ± 2.49	76.09 ± 2.61	75.12 ± 3.3	75.55 ± 2.53	72.87 ± 3.04	71.59 ± 3.78	70.28 ± 3.89	71.33 (68.37–73.62)	< 0.001
**Parafoveal CVD**	70.86 ± 4.46	70.31 ± 4.8	72.16 ± 2.99	73.22 ± 4.38 )	73.25 ± 3.15	71.14 ± 3.09	70.01 ± 4.12	68.37 ± 4.97	67.56 ± 4.43	< 0.001
**CVI**	0.7 ± 0.02	0.69 ± 0.24	0.69 ± 0.03	0.7 ± 0.02	0.7 ± 0.02	0.71 ± 0.02	0.7 ± 0.03	0.7 ± 0.03	0.69 ± 0.03	0.225
**FAZ**	0.27 ± 0.1	0.28 ± 0.08	0.28 ± 0.11	0.23 ± 0.09	0.24 ± 0.09	0.3 ± 0.13	0.26 ± 0.07	0.29 ± 0.1	0.32 ± 0.12	0.016

**Table 2:** Retinal thickness of foveal and parafoveal retina in healthy subjects in 8 age groups. Abbreviations: WRT, whole retinal thickness; IRT, inner retinal thickness; MRT, middle retinal thickness; ORT, outer retinal thickness

		Group 1	Group 2	Group 3	Group 4	Group 5	Group 6	Group 7	Group 8	
Groups	Total (435 Eyes)	< 10 (61 Eyes)	10–20 (66 Eyes)	20–30 (63 Eyes)	30–40 (54 Eyes)	40–50 (47 Eyes)	50–60 (57 Eyes)	60–70 (65 Eyes)	> 70 (22 Eyes)	*P*
**Foveal WRT**	257.57 ± 19.48	246.68 ± 12.31	253 ± 18.84	263.76 ± 18.03	259.67 ± 16.75	256.2 ± 23.46	267.55 ± 19.7	256.95 ± 20.23	257.47 ± 18.54	< 0.001
**Parafoveal WRT**	330.99 ± 15.48	327.91 ± 12.24	331.24 ± 13.11	335.09 ± 16.55	333.57 ± 13.57	330.16 ± 18.6	334.81 ± 17.75	325.89 ± 14.79	327.58 ± 15	0.107
**Foveal IRT**	47.96 ± 8.54	45.81 ± 6.65	46.76 ± 7.31	51.98 ± 8.37	49.17 ± 8.49	47.28 ± 11.29	50 ± 7.35	45.69 ± 9.22	45.84 ± 6.42	0.007
**Parafoveal IRT**	112.5 ± 8.68	111.21 ± 5.99	113.02 ± 7.61	116.02 ± 8.23	114.3 ± 6.98	111.65 ± 10.2	114.52 ± 9.9	108.04 ± 9.2	109.83 ± 8.41	0.002
**Foveal MRT**	44.53 ± 7.41	42.72 ± 6.2	43.31 ± 5.9	45.19 ± 5.96	43.57 ± 8.06	44.78 ± 7.75	48.68 ± 8.02	43.94 ± 8.33	44.04 ± 8.24	0.04
**Parafoveal MRT**	70.84 ± 5.54	73.19 ± 5.07	70.98 ± 5.25	69.44 ± 5.04	70.04 ± 5.73	70.34 ± 5.09	71.95 ± 5.21	69.46 ± 5.77	72.12 ± 7.3	0.006
**Foveal ORT**	170.07 ± 11.03	163.14 ± 10.07	168.06 ± 10.65	171.58 ± 10.08	171.8 ± 11.08	169.14 ± 11.08	173.85 ± 10.21	172.22 ± 10.19	172.49 ± 13.11	0.001
**Parafoveal ORT**	149.48 ± 9.28	145.05 ± 9.32	148.88 ± 8.37	151.39 ± 8.62	151.08 ± 9.41	149.82 ± 9.46	150.51 ± 9.19	150.43 ± 8.91	147.99 ± 11.14	0.101
**SFCT**	240.57 ± 44.14	263.33 ± 52.18	234.75 ± 48.89	243.16 ± 35.36	250.56 ± 33.25	239.13 ± 53.17	251.91 ± 40.31	216.93 ± 48.89	213.98 ± 47.88	0.055

**Table 3:** Comparison of foveal and parafoveal vascular densities between males and female in different age groups. Abbreviations: SVD: superficial vascular density; DVD, deep vascular density; CVD, choroidal vascular density; CVI, choroidal vascular index; FAZ, foveal avascular zone

		Foveal SVD	Parafoveal SVD	Foveal DVD	Parafoveal DVD	Foveal CVD	Parafoveal CVD	FAZ	CVI
**Total**	**Female**	18.4 ± 7.25	50.83 ± 3.41	33.22 ± 7.72	54.44 ± 3.45	72.46 ± 4.14	70.02 ± 4.41	0.3 ± 0.11	0.7 ± 0.02
	**Male**	22.22 ± 6.31	51.41 ± 3.04	35.86 ± 6	52.62 ± 3.37	74.05 ± 3.84	71.53 ± 4.46	0.25 ± 0.09	0.7 ± 0.03
	**P**	< 0.001	0.168	0.007	< 0.001	0.003	0.012	0.001	0.3
**< 10**	**Female**	17.08 (16.57–19.89)	49.81 (47.78–53.36)	36.24 (32.73–37.37)	57.6 (53.64–59.43)	77.68 (72.31–80.4)	68.21 (61.4–74.9)	0.26 (0.25–0.33)	0.68 ± 0.003
	**Male**	20.77 ± 5.87	52.6 ± 2.26	37.13 ± 6.24	55.38 ± 3.24	76.78 ± 2.03	70.82 ± 4.14	0.27 ± 0.08	0.69 ± 0.3
	**P**	0.134	0.06	0.418	0.392	0.945	0.503	0.771	0.038
**10–20**	**Female**	15.68 ± 5.8	52.33 ± 1.95	32.27 ± 7.14	55.54 ± 1.96	75.58 ± 2.66	71.05 ± 2.55	0.32 ± 0.1	0.68 ± 0.004
	**Male**	18.95 (17.47–24.8)	51.37 ± 1.78	37.05 ± 6.08	55.45 ± 2.54	76.53 ± 2.54	73.89 (71.96–75.01)	0.24 ± 0.1	0.69 ± 0.3
	**P**	0.035	0.127	0.098	0.915	0.357	0.051	0.06	0.07
**20–30**	**Female**	23.23 ± 8.42	52.09 ± 2.99	38.25 ± 8.33	55.89 ± 2.44	74.36 (72.9-75.39)	72.12 (68.84–74.27)	0.23 ± 0.11	0.71 ± 0.02
	**Male**	26.22 ± 3.98	52.72 ± 2.49	38.86 (32.13–40.54)	53.16 ± 2.97	76.35 (75.55–77.65)	75.09 ± 2.07	0.24 ± 0.08	0.70 ± 0.02
	**P**	0.272	0.525	0.706	0.003	0.036	0.005	0.913	0.58
**30–40**	**Female**	23.35 ± 5.39	53.1 ± 2.27	37.08 ± 6.17	56.2 ± 2.59	74.74 ± 3.29	71.86 ± 3.63	0.26 ± 0.09	0.7 ± 0.02
	**Male**	26.78 ± 5.47	52.79 ± 2.37	36.55 ± 4.86	52.8 ± 3.04	76.15 ± 1.57	74.3 ± 2.27	0.22 ± 0.08	0.71 ± 0.02
	**P**	0.079	0.682	0.797	< 0.001	0.13	0.021	0.213	0.258
**40–50**	**Female**	16.24 ± 7.06	51.44 ± 2.93	29.22 ± 8.43	54.58 ± 2.13	72.29 ± 3.06	71.32 ± 3.86	0.36 ± 0.13	0.70 ± 0.01
	**Male**	22.74 ± 7.21	51.37 ± 2.13	35.61 ± 7.59	51.7 ± 2.77	73.39 ± 2.97	70.98 ± 2.26	0.25 ± 0.11	0.71 ± 0.03
	**P**	0.014	0.942	0.036	< 0.001	0.251	0.753	0.022	0.66
**50–60**	**Female**	19.12 ± 6.57	49.28 ± 4.22	35.04 ± 5.2	53.7 ± 3.67	71.67 (69.56–73.64)	71.98 (67.92–73.91)	0.27 ± 0.07	0.72 ± 0.004
	**Male**	20.86 ± 5.34	51.71 ± 2.88	35.64 ± 4.28	52.08 ± 2.32	72.15 ± 3.32	69.76 ± 3.84	0.25 ± 0.07	0.69 ± 0.03
	**P**	0.46	0.041	0.739	0.146	0.279	0.68	0.528	0.001
**60–70**	**Female**	15.68 ± 7.24	49.3 ± 3.29	30.63 ± 7.07	53.79 ± 3.19	70.47 ± 3.49	68.59 ± 3.96	0.32 ± 0.1	0.70 ± 0.03
	**Male**	19.01 ± 5.37	48.56 ± 3.33	34.64 ± 6.58	50.43 ± 3.52	70.08 ± 4.31	68.13 ± 5.91	0.26 ± 0.08	0.71 ± 0.05
	**P**	0.091	0.437	0.059	0.001	0.729	0.766	0.038	0.80
**> 70**	**Female**	16.78 ± 5.86	49.04 ± 3.49	31.34 ± 8.14	50.65 ± 4.87	71.42 (67.24–73.59)	67.21 ± 4.5	0.32 ± 0.12	0.72 ± 0.02
	**Male**	13.86 ± 5.17	48.69 ± 4.89	27.76 ± 5.45	49.47 ± 3.04	70.71 ± 3.67	68.47 ± 4.45	0.34 ± 0.12	0.67 ± 0.01
	**P**	0.187	0.86	0.201	0.46	0.759	0.531	0.602	0.001

**Table 4:** Comparison of the retinal thickness of foveal and parafoveal between males and females in different age groups. Abbreviations: WRT, whole retinal thickness; IRT, inner retinal thickness; MRT, middle retinal thickness; ORT, outer retinal thickness

		Foveal WRT	Foveal IRT	Foveal MRT	Foveal ORT	Parafoveal WIRT	Parafoveal IRT	Parafovea MRT	Parafoveal ORT
**Total**	**Female**	251.55 ± 18.25	45.71 ± 8.8	43.61 ± 7.57	167.28 ± 10.28	325.78 ± 14.26	110.52 ± 8.47	70.01 ± 5.46	147.03 ± 7.9
	**Male**	261.9 ± 19.22	49.57 ± 7.99	45.18 ± 7.24	172.07 ± 11.14	334.73 ± 15.26	113.93 ± 8.56	71.44 ± 5.53	151.24 ± 9.8
	**P**	< 0.001	0.001	0.097	< 0.001	< 0.001	0.002	0.03	< 0.001
**< 10**	**Female**	245.23 ± 11.5	43.6 ± 7.05	41.55 ± 6.22	165.16 ± 9.54	327.99 ± 14.53	110.92 ± 8.6	73.24 ± 5.57	145.53 ± 8.94
	**Male**	247.2 ± 12.67	46.59 ± 6.4	43.14 ± 6.21	162.42 ± 10.26	327.88 ± 11.51	111.32 ± 4.87	73.17 ± 4.94	144.88 ± 9.54
	**P**	0.663	0.264	0.506	0.461	0.983	0.894	0.966	0.847
**10–20**	**Female**	250.5 ± 20.75	45.77 ± 6.94	44.31 ± 7.43	165.96 ± 10.21	335.9 ± 15.48	115.68 ± 9.33	72.34 ± 4.91	149.21 ± 6.74
	**Male**	254.24 ± 17.93	47.26 ± 7.52	42.81 ± 4.98	169.12 ± 10.82	328.91 ± 11.22	111.68 ± 6.28	70.31 ± 5.33	148.71 ± 9.15
	**P**	0.6	0.559	0.508	0.387	0.169	0.185	0.189	0.848
**20–30**	**Female**	260.2 (235.3–279)	49.87 ± 10.45	45.33 ± 8.01	166.13 ± 10.54	324.99 ± 11.4	111.64 ± 5.07	67.25 ± 4.57	145.9 (143.4-150.4)
	**Male**	267.17 ± 14.52	52.97 ± 7.14	45.13 ± 4.84	174.11 ± 8.89	339.78 ± 16.56	118.05 ± 8.65	70.47 ± 4.96	153.12 ± 8.56
	**P**	0.142	0.378	0.936	0.017	0.002	0.007	0.038	0.054
**30–40**	**Female**	257.49 ± 15.4	47.81 ± 7.92	43.28 ± 7.63	171.41 ± 11.6	330.75 ± 12.88	113.26 ± 5.27	69.69 ± 5.61	149.75 ± 7.9
	**Male**	262.39 ± 18.27	50.88 ± 9.02	43.93 ± 8.72	172.3 ± 10.61	337.1 ± 13.84	115.6 ± 8.6	68.4 (66.6–72.7)	152.74 ± 10.95
	**P**	0.419	0.336	0.821	0.811	0.174	0.369	0.677	0.384
**40–50**	**Female**	238.22 ± 15.11	38.3 (33.6–43.7)	41.81 ± 7.86	157.6 (154.8-168.7)	317.22 ± 10.88	105.47 ± 8.26	69.06 ± 3.77	142.5 (139.8-144.2)
	**Male**	269.52 ± 19.34	52.54 ± 10.39	46.98 ± 7.01	174.88 ± 8.92	339.75 ± 17.35	116.23 ± 9.12	71.3 ± 5.77	154.07 ± 8.9
	**P**	< 0.001	0.001	0.076	< 0.001	< 0.001	0.001	0.162	0.001
**50–60**	**Female**	259.59 ± 13.54	47.97 ± 7.16	46.55 ± 5.8	169.98 ± 7.46	326.98 ± 10.95	110.7 ± 7.48	71.81 ± 5.44	146.64 ± 5.78
	**Male**	273.33 ± 21.57	51.47 ± 7.25	50.23 ± 9.08	182.4 (168-186.3)	340.5 ± 19.64	117.3 ± 10.6	72.05 ± 5.11	157.1 (145.2-161.5)
	**P**	0.03	0.182	0.144	0.041	0.015	0.042	0.896	0.018
**60–70**	**Female**	249.17 ± 20.19	44.14 ± 10.82	43.13 ± 9.22	166.9 ± 9.3	319.96 ± 15.47	106.98 ± 10.43	68.02 ± 5.57	146.75 ± 8.58
	**Male**	265.48 ± 16.77	47.38 ± 6.86	44.83 ± 7.29	178.06 ± 7.69	332.41 ± 10.92	109.19 ± 7.63	71.04 ± 5.66	154.46 ± 7.49
	**P**	0.007	0.266	0.517	< 0.001	0.004	0.445	0.064	0.003
**> 70**	**Female**	251.69 ± 14.48	43.9 (42.8–46.5)	42.08 ± 5.88	169.08 ± 12.48	322.58 ± 12.92	110.04 ± 7.25	70.34 ± 5.61	144.55 ± 9.86
	**Male**	272.88 ± 20.57	46.82 ± 6.33	49.25 ± 11.7	181.58 ± 10.86	340.92 ± 12.3	109.28 ± 11.78	76.85 ± 9.61	157.15 ± 9.54
	**P**	0.003	0.615	0.088	0.027	< 0.001	0.868	0.156	0.013

### The relationship between VD and RT and age

The overall foveal SVD, DVD, and CVD were 20.51 ± 7.00, 34.68 ± 6.94, and 73.34 ± 4.05%, respectively. Also, the overall SVD, DVD, and CVD of parafovea were quantified as 51.15 ± 3.22, 53.43 ± 3.52, and 70.86 ± 4.46%, respectively. The mean foveal SVD and DVD and parafoveal SVD had the highest level of density between the 20–40 years and tended to decrease to lowest point by the seventies. Foveal CVD, and parafoveal DVD had a decreasing order during the lifespan. Parafoveal CVD had increased up to 30–40 years and then the decreasing order started after 40 years old up to the last group of age (Table 1). There was no significant difference in CVI between age groups; all ages showed a value of around 70% (*P* = 0.225). FAZ revealed relatively minor undulation in the size curve across age groups, with less amount between the ages 20 to 40 (*P* = 0.01).

The values of RT, including full retina thickness, IRT, MRT, and ORT of fovea and parafovea, were calculated. The whole foveal thickness, and foveal IRT, MRT, and ORT were 257.57 ± 19.48, 47.96 ± 8.54, 44.53 ± 7.41, and 170.07 ± 11.03 μm, respectively. Also, the whole parafoveal RT, and parafoveal IRT, MRT, and ORT were 330.99 ± 15.48, 112.5 ± 8.68, 70.84 ± 5.54, and 149.48 ± 9.28 μm, respectively. Like VD, a statistically meaningful relationship was detected between age and all evaluated RT parameters (Tables 1 and 2). At the foveal and parafoveal part, the whole RT was thickest at ages between 20 and 40. IRT had the thickest amount at age twenties in the fovea and parafovea. In the foveal area, the thickest MRT was in the fifties, and the parafoveal area at the 10–20-year group. ORT was thickest in the fifties in both foveal and parafoveal areas (Table 2).

### The relationship between VD and RT and gender

The difference in retinal VD between genders was more noticeable in males across all age groups up to 70 years, particularly in the foveal area (Table 3). Specifically, the VD in the foveal area was measured at 22.22 ± 6.31% in males and 18.4 ± 7.25% in females. Apart from parafoveal SVD, all other five parameters (foveal SVD, DVD, and CVD, parafoveal DVD, and parafoveal CVD) showed statistically significant differences between males and females. Males consistently demonstrate elevated VD levels compared to females across these parameters, except for parafoveal DVD, which is higher in females.

Notably, no significant gender differences in VD were observed in age groups below 10 and above 70 years. Remarkably, Table 3; Fig. 1 illustrate the most significant gender-based variations in foveal SVD at the 10–20 and 40–50 age groups and DVD occurs in the 10–20 age groups. In the parafoveal area, SVD differed between the genders at 50–60 years and for DVD at the 20–30, 30–40, 40–60, and 60–70 years of ages. The main differences in CVI were at extremes of age groups and at 50–60 years of age which was higher in females at older ages (Fig. 3). FAZ showed differences in the 40–50 and 60–70 age groups showing reciprocally more size in females just at these periods.

When considering the entire foveal RT, males had a measurement of 261.9 ± 19.22 μm while females had a measurement of 251.55 ± 18.25 μm (*P* < 0.001) (Fig. 2). In terms of the whole parafoveal RT, males had a measurement of 334.73 ± 15.26 μm, whereas females had a measurement of 325.78 ± 14.26 μm (*P* < 0.001). Generally, RT across all layers was lower in females compared to males (*P* < 0.05). There was no difference in MRT between the foveal and parafoveal areas in both groups throughout the studied lifespans (Table 4).

## Discussion

Our study found a significant relationship between age and retinal VD as well as layered RT. The overall trend of vascular densities in the foveal and parafoveal areas is a gradual decrease. However, the superficial capillary plexus (SCP) shows an upward slope until the age of 40, after which it rapidly declines in density with advancing age. Males had higher VD compared to females across all parameters except for parafoveal DVD. Like VD, age also showed a significant relationship with all total and segmental and layered RT parameters. The foveal RT chart displayed a gradual incline, while the RT in the parafoveal region exhibited an upward trend until the age of 20–30. Afterward, the slope leveled off and remained constant. RT across all layers was lower in females compared to males.

Understanding the normal values for RT and VD by OCT and OCTA helps in quickly spotting any potential issues or abnormalities [10]. We observed that at the foveal and parafoveal part, the entire RT was thicker at ages between 20 and 40. The foveal and parafoveal IRT showed the greatest level of thickness in the 20–30 age group. In the foveal area, the thickest MRT was in the fifties, and in the parafoveal area it was at age 10–20. ORT was thickest in the fifties in both foveal and parafoveal areas. The foveal and parafoveal regions exhibit the highest thickness levels between the ages of 20 and 60.

Inconsistent findings have emerged from research examining the thickness of individual retinal layers at the fovea in relation to aging. Ooto et al. revealed an augmentation in foveal RT, alongside a significantly heightened thickness of both photoreceptor outer segments and the OPL and ONL with advancing age [11]. Conversely, Demirkaya et al. identified a reduction in the retinal outer segment layer with increasing age and no alteration in the other layers of the fovea [12]. Bafiq et al. showed that the RT exhibited an inverse relationship with age, with a decrement of 0.67µmm per annum beyond the 1 mm circumference of fovea. The rates of decline in thickness differed between the inner and outer layers of the retina [13]. The process of aging has been demonstrated to be linked with the loss of neurons and glial cells in the inner layer of the retina. Examination of histological studies revealed age-related losses of 0.3–0.6% of retinal neurons per year [14]. Eriksson and Alm [15] reported a negative correlation between RT in all nine ETDRS subsegments and age. Similarly, Manassakorn et al. [16] discovered a significant association between age and macular thickness in all ETDRS areas except for the center.

Our results showed the thickest IRT in the twenties and its slowly decrease during aging. In accordance, Cavallotti et al. [17] found that RT decreases significantly with age in a histologic study. They reported a decrease in the number of ganglion cells with increased age. It appears that these cells are more susceptible to age-related loss than other retinal cells. The number of retinal capillaries, intercellular connections, cellular processes, and synaptic bodies also decrease markedly with aging. A study by Ono and colleagues [18] gives inconclusive data about the thickness of the retinal nerve fiber layer (RNFL) and does not provide good evidence of a significant correlation between RNFL and age. Nonetheless, Funaki and associates [19] reported that RNFL thickness decreases significantly with aging.

The increase in ORT in our study is not in agreement with the study of Gao and Hollyfield [20] who reported substantial progressive loss of neurons (photoreceptors) with aging. They reported that during the second to ninth decade, there was a rate of loss of equatorial RPE cells of approximately 14 cells/mm^2^ per year, according to their findings. We think that outer retinal thickening in our study could be due to the structural interstitial cells in the outer retina as well as the thickening of the photoreceptor’s outer segments due to structural or functional decrease in the dissolution and resorption of the photoreceptors’ outer segments. Actual changes in the overall size of the photoreceptor cells could be another culprit [17–21].

We found a discrepancy in the foveal area and parafoveal area regarding the thickness of MRT in the life course, as the thickest foveal MRT was in the fifties, and the thickest parafoveal MRT was in the 10–20-year group. It could be hypothesized that thickening occurs in the OPL at the fovea during time due to remodeling of foveal and parafoveal areas due to the plasticity of the retina at these areas. This layer contains the synapses between the axons of photoreceptor cells and the dendrites of intermediate neurons (bipolar and horizontal cells). An alternative hypothesis is to increase the neural or axonal content or interfibrillar glial tissue.

The other finding of our study was the correlation between all RT parameters and gender. All RT parameters were found to be significantly higher in males. However, among individuals up to 40 years, there was no significant disparity observed between males and females. Prior studies have indicated the disparities between genders and RT [22, 23]. Findings from various ethnic populations show that women have a thinner retina in the fovea and parafoveal area compared to men. Bafiq and colleagues found that females have lower RT values in both the central sub-field and the inner retinal layers compared to males. Specifically, females had a 16 μm less thickness in central sub-field thickness and a 6 μm less in IRT [13]. Notably, studies on children have shown that boys tend to have a thicker macula than girls, although some studies have reported no gender-based differences [24–26]. Furthermore, in another study no significant discrepancy in mean foveal thickness between men (204 μm; range, 154–232 μm) and women (207 μm; range, 173–252 μm) was detected [7]. Collectively, our findings are in line with the concept of the impact of gender on RT. The decreased thickness of the fovea in female could explain more susceptibility of them to develop macular holes. It has been proposed that macular hole formation begins with thinning of the fovea. However, further research is needed to establish the connection between hormonal effects and RT.

In the present investigation, we observed a statistical significance of the relationship between age and all VD parameters. All VD metrics indicated a decreasing tendency after the age of forty. Consistent with our results a study in 224 eyes of 112 individuals (12–67 years) showed that the VD of parafoveal and foveal areas reduced with age, but mainly in patients over 50 years old. This study concluded that, for each year of increase in age, VD of the SCP and DCP in the macula decreases by 0.32%, and 0.48%, respectively [27].

Wei et al. enrolled individuals ranging from the ages of 18 to 82 years and categorized them into four groups according to age. Their investigation revealed a negative correlation between age and retinal VD [28]. In a study by Wang et al. [29], a greater density of SCP and DCP were correlated with a younger age. Similarly, in an investigation by Yu et al. [32 − 30], on a sample of 45 healthy Chinese, observed that the mean parafoveal flow index and the mean vascular area density notably decline with age. Park and colleagues [31] showed that the densities of the retinal and choroidal capillaries, as well as the RT and CT, exhibit a tendency to diminish as individuals age, particularly after the age of 40, which is consistent with our study. Also, following age 40, the VD at parafoveal region diminished with a reduction in RT. Previously, Ding and colleagues [32] have documented a notable reduction in subfoveal CT in individuals aged 60 years and above. Additionally, Ito and colleagues [33] have reported that reductions in choroidal circulation are particularly noticeable in individuals aged 50 years and above.

Gender variations were observed in the VD across different regions of the retina among males and females. Males generally had higher VD than females, except for the VD at parafoveal area. Previous research has shown that males tend to have higher density in the SCP, while females have higher density in the DCP [29]. Also, some studies have reported higher SCP density in males [34]. Additionally, it has been noted that females, especially those over 60 years old, may have higher VD, possibly due to a slower rate of aging of blood vessels in women according to Coscas et al. [35]. In our study, females had higher parafoveal DVD.

Regarding CVI, a study by Ruiz-Medrano et al. found a significant difference in CVI levels between individuals over 18 years old and those under 18 [36]. In contrast to our study, another study, on 106 healthy eyes in individuals aged 21 to 78 years old, found that the CVI decreased as people got older. The decline in CVI was noticeable in individuals aged 33 to 43, showing a decrease of 0.7 to 2.7% per decade [37]. Our findings indicate that there is no significant variation in CVI among different age groups. The most noticeable disparities in CVI were observed at the youngest and oldest age groups. We noticed a higher amount of CVI among females in the 50–60 age group.

Our investigation possesses various advantages of a broad spectrum of age, utilization an imaging modality exhibiting good resolution of capillaries, and integration of an automated methodology for analysis. However, there are several limitations to our work. Our study used a 3 × 3 mm OCTA scan, which only roughly cover the parafoveal area. Limited by small sample size and nonlongitudinal design, our study calls for future research with long follow-up to assess repeatability of macular layer measurements of the same participants by aging. Further, understanding peripheral retinal regions and changes of macular morphology over time requires wide-field scanning methods. The influence of ethnic diversity on VD has not been assessed in this investigation. The clinical significance of the findings from this study remains to be established; however, considering the normal reduction in vascular density VD and RT after the age of 40, particular attention should be directed towards the prevalence of age-related retinal as macular holes and age-related macular degeneration, as well as specific forms of retinal dystrophies. These observations warrant validation through histological studies.

In conclusion, age was found to be significantly related to retinal VD and layered RT. Vascular densities in the foveal and parafoveal regions generally decreased, although the SCP increased until age 40 before rapidly declining. Males had higher VD except for parafoveal DVD. Foveal RT increased gradually, while parafoveal RT increased until 20–30 and then leveled off. Females had lower RT than males across all layers.

## Data Availability

No datasets were generated or analysed during the current study.
